# Missing in space: an evaluation of imputation methods for missing data in spatial analysis of risk factors for type II diabetes

**DOI:** 10.1186/1476-072X-13-47

**Published:** 2014-11-20

**Authors:** Jannah Baker, Nicole White, Kerrie Mengersen

**Affiliations:** Queensland University of Technology School of Mathematical Sciences, Brisbane, Australia; Cooperative Research Centres for Spatial Information, Melbourne, Australia

**Keywords:** Imputation, Missing, Spatial, Prevalence, Diabetes

## Abstract

**Background:**

Spatial analysis is increasingly important for identifying modifiable geographic risk factors for disease. However, spatial health data from surveys are often incomplete, ranging from missing data for only a few variables, to missing data for many variables. For spatial analyses of health outcomes, selection of an appropriate imputation method is critical in order to produce the most accurate inferences.

**Methods:**

We present a cross-validation approach to select between three imputation methods for health survey data with correlated lifestyle covariates, using as a case study, type II diabetes mellitus (DM II) risk across 71 Queensland Local Government Areas (LGAs). We compare the accuracy of mean imputation to imputation using multivariate normal and conditional autoregressive prior distributions.

**Results:**

Choice of imputation method depends upon the application and is not necessarily the most complex method. Mean imputation was selected as the most accurate method in this application.

**Conclusions:**

Selecting an appropriate imputation method for health survey data, after accounting for spatial correlation and correlation between covariates, allows more complete analysis of geographic risk factors for disease with more confidence in the results to inform public policy decision-making.

**Electronic supplementary material:**

The online version of this article (doi:10.1186/1476-072X-13-47) contains supplementary material, which is available to authorized users.

## Background

Spatial analysis is being used increasingly to identify geographic risk factors associated with disease and areas at high excess risk of disease beyond what would be expected given the prevalence of these risk factors. Many geographic risk factors are modifiable and amenable to health promotion programmes, thus spatial analysis can provide useful information to inform resource allocation and public policy decisions. Maps of spatial models have been useful for highlighting differential risk across regions. They are particularly useful for small area estimation, since the accuracy and precision of estimates based on small counts in a region can be improved by “borrowing strength” from estimates in neighbouring regions [1]. Bayesian models are particularly well suited to spatial modelling since the information provided by neighbouring regions can be naturally represented as priors [2].

Routinely collected survey data can provide useful information about the distribution of covariates at a regional level, but frequently a problem with such data is the presence of missing covariate information. Often the data are spatially correlated and/or there are correlations between covariates. In these cases, imputation of missing data with plausible values allows inferences to be made about outcomes and covariates using statistical methods suited to complete data. Several methods of imputation are available and it is important to select the one best suited to a particular dataset.

In this paper, we address this challenge by considering a case study of geographic risk factors associated with type II diabetes (DM II).

The prevalence of DM II is increasing worldwide, with a report from Diabetes UK reporting a “state of crisis” in diabetes care [3]. Diabetes is reported to affect 11.3% of the US and 4.45% of the UK adult population, of which DM II accounts for 90-95% of cases [3, 4]. Diabetes is reported to be the leading cause of renal failure, nontraumatic lower-limb amputation, and new cases of blindness, the major cause of heart disease and stroke, and the seventh leading cause of death in the US [4].

Despite the rising shortage of service provision for DM II, there is evidence that DM II is preventable in 60% cases with lifestyle change and/or medications [5]. Thus long-term consequences of DM II can be prevented through early detection and management of glycaemic control and cardiovascular risk factors [6]. Evidence shows that DM II is associated with both environmental and individual factors [7]. Therefore, analysis of geographic differences in DM II incidence may provide important information for more targeted intervention and management, and hence may be useful for informing resource allocation decisions.

Demographic and lifestyle factors associated with increased risk of developing DM II include male gender, increasing age, increasing BMI, increasing waist:hip ratio, indicators of low socio-economic status, sedentary lifestyle, physical inactivity, smoking history, and low levels of fruit and vegetable consumption [8–12]. In addition, spatial studies of DM II that aim to describe changes in DM II outcomes over a set of neighbouring regions have shown DM II to be associated with deprivation [12], socioeconomic status [9, 11, 13, 14] and smoking prevalence [11] at a regional level. However, these studies have only been conducted in a very limited number of countries to date. Moreover, there is a lack of spatial studies examining the association of DM II relative risk (RR) with the distribution of other candidate lifestyle factors such as overweight/obesity, physical activity levels and fruit and vegetable consumption at a regional level.

Spatial studies examining DM II outcomes over regions have been developed in the US, England and Europe [7, 9, 11–18]. Spatial models estimated by Bayesian methods have successfully been used to model several diseases including DM II (Liese, Chaix, Congdon, Bayesian GLMMs), anaemia [19], dental caries [20], leprosy [21], multiple sclerosis [22], cancer incidence and mortality risk [23–25], malaria [26–28], and childhood leukaemia and lymphoma [29]. In our case study, we fit Bayesian spatial models to DM II prevalence data across Queensland regions, accounting for significant missing data.

This study has four objectives: a) to trial and select an appropriate imputation method to account for missing survey data from a number of relevant choices, b) to examine geographic disparities in DM II RR in Queensland, b) to identify areas with high DM II RR in this region, and d) to identify environmental risk factors for DM II RR at a regional level.

## Methods

For clarity, we first introduce the case study, then consider imputation methods, and finally evaluate these alternative methods in the context of the case study.

### Case study

This case study examines disparities in the RR and relative excess risk (RER) of DM II across 71 Queensland LGAs, accounting for seven geographic lifestyle factors, after selection of the most appropriate imputation method out of three alternative methods. RR is defined as the ratio of the estimated risk in a particular LGA to the mean estimated risk across all LGAs; thus LGAs with a larger RR are estimated to be more at risk for DM II prevalence than LGAs with smaller RRs. RER is defined as the estimated excess risk for DM II prevalence in a particular LGA after taking into account the effect of lifestyle covariates in that region. Thus LGAs with a larger RER have unexplained higher risk for DM II prevalence than would be expected and may benefit more from programmes for early detection and management of DM II.

### Sources of data

Our analysis of the region-level determinants of DM II relative risk relied on three databases, briefly described below.

1. The National Diabetes Services Scheme (NDSS) database for 2011 diabetic notification data [30]. The NDSS delivers diabetes-related products, information and support services to almost 1.1 million Australian with diabetes and monitors the prevalence of diabetes including DM II across regions in Australia. This database also contains 2011 data originally from the Australian Bureau of Statistics (ABS) for a) socioeconomic status (SES) measured by average income scored 1–10 (1 indicating lowest and 10 indicating highest income decile across Australia) and b) proportion over the age of 45 years for the general population in each LGA in Queensland, which were used as covariates in this case study.
2. The 2011 census information from the ABS for estimated resident population (ERP) per LGA [31]. The ABS collects and publishes census data and monitors population counts across regions in Australia.
3. The Queensland self-reported health status 2009–2010: Local Government Area summary report weighted by age and gender distribution [32]. This survey estimates the prevalence of key population health indicators for those aged 18 years and older for each Queensland LGA based on self-report, including body mass index (BMI) from self-reported height and weight, proportion of daily smokers, proportion with insufficient physical activity for health benefit, adequate fruit intake (2+ serves/day), and adequate vegetable intake (5+ serves/day). The proportion overweight or obese in each LGA, defined as BMI ≥ 25kg/m^2^, was estimated from self-reported height and weight.

The survey provides a total of 16,530 completed computer-assisted telephone interviews across Queensland, with a response rate of 56.7% in 2009 and 64.5% in 2010. The telephone numbers selected for this survey were reportedly sourced by random digit dialling (RDD) using a specific sample frame from the Association of Market and Social Research Organisations RDD sample database. Data are reported for LGAs that had a sample of 60 or more completed interviews (Brisbane LGA had the largest number of interviews at 2,561). Data are not reported from this survey for 28 LGAs with a sample size smaller than 60 due to potential inaccuracy of estimates.

The reported overall prevalence of DM II across all Queensland LGAs from NDSS data were combined with ERP data to compute estimated counts for each LGA. Three island LGAs (Mornington, Palm Island and Torres Strait Island) were excluded, leaving 71 Queensland LGAs included in this spatial analysis.

### Ethical Statement

The QUT University Human Research Ethics Committee assessed this research as meeting the conditions for exemption from HREC review and approval in accordance with section 5.1.22 of the National Statement on Ethical Conduct in Human Research (2007). Exemption number: 1400000354 QV reference no.: 44305.

### Spatial model

Multivariable models including all seven lifestyle covariates were fitted to the DM II prevalence data.

Bayesian generalised linear mixed models (GLMMs) using Markov chain Monte Carlo (MCMC) were used to model RR and prevalence across regions. Two general models were considered: a Binomial model and a Poisson model. The Binomial GLMMs took the form:
1

where for region *i*, *Y*_*i*_ is the observed number of DM II cases, *p*_*i*_ is the estimated prevalence of DM II, and *n*_*i*_ is the estimated resident population. *α* is a fixed intercept, ***β*** is a vector of coefficients, and *x*_*i*_ is the *i* th row of the design matrix **X**, containing covariate data for region *i*. The uncorrelated error for region *i* is denoted *U*_*i*_, and *S*_*i*_ is the correlated spatial error based on neighbourhood information; this is described in more detail below. Separating the residual error into spatial (*S*_*i*_) and non-spatial (*U*_*i*_) components provides an indication of how much variation in DM II prevalence can be attributed to the effect of geographical region, after accounting for the effect of the covariates.

The Poisson GLMMS took the general form:
2

where for region *i*, *Y*_*i*_ is the reported number of DM II cases, λ_*i*_ is the estimated RR of DM II, *E*_*i*_ is the expected count and the other terms are as defined above. The expected DM II count in each region was computed as a product of the average DM II prevalence across Queensland (internal to the dataset) and the ERP for each LGA.

The intrinsic conditional autoregressive (CAR) prior, first described by Besag in 1974, were fit to the spatially correlated residual terms in equations (1) and (2) [33]. This prior assumes that the value of *S*_*i*_ is normally distributed around the values of *S*_*i*_ in the neighbouring regions, ie:
3

where *μ*(*s*_*k*_) is average correlated random effect for the neighbours of region *i*, *m*_*i*_ is the number of such neighbours, and  is the conditional variance of *S*[34]. A neighbour is defined as any region adjacent in space to region *i*. It can be seen that this type of prior induces a form of local smoothing across regions, where the degree of smoothing is controlled by the spatial correlation between regions [1]. An advantage of the CAR model is that the conditional dependencies can be modelled as part of the usual Bayesian MCMC analysis [34].

Results are reported from a baseline model, to which models with other choices of priors were compared in sensitivity analysis. The baseline model has CAR priors fit to both correlated random effects, *V*_*i*_, and to covariate data ***X***, and Gamma(1,0.01) priors for the precisions of *U*_*i*_.

For both Binomial and Poisson models, RER was computed for each LGA based on residual error after accounting for the variation attributed to the effects of covariates as follows:


The RER provides an indication of regions where the estimated risk is greater or smaller than would be expected after accounting for the influence of lifestyle risk factors in that region.

Estimation of model parameters and mapping of results was performed using R 2.15.0 and WinBUGS 14 [35, 36]. Results presented for each model are based on 100,000 iterations, following a burn-in of 50,000 iterations. The number of iterations and burn-in used in each model were selected based on the appearance of trace plots for parameters. Covariates representing proportion over 45 years of age, proportion overweight or obese, proportion of daily smokers, proportion with insufficient physical activity, proportion with adequate fruit intake and proportion with adequate vegetable intake were centred around their mean to improve model convergence. Correlations between covariates were assessed using Pearson’s R. Model fit was compared between models using deviance information criteria (DIC) [37]. DIC consists of two components, a term that measures goodness of fit () and a term that penalises models for the number of parameters (*p*_*D*_), thus favouring simpler models.


where  is expected deviance over the course of MCMC, *T* is the total number of iterations, *D*(*y*, *θ*) is the deviance of the unknown parameters of the model *θ*, y are the data, *p*(*y*|*θ*) is the likelihood function of observing the data given the model, and *C* is a constant that cancels out in calculations comparing different models. The expectation,  is a measure of how well the model *θ* fits the data – the smaller the value of , the better the fit. Smaller values of DIC are indicative of an improved model.

In addition to multivariable models, the effect of each covariate individually on DM II RR was evaluated with univariate models. It was also considered that SES may potentially be a more distal factor influencing levels of the other lifestyle covariates: thus, potential mediation between SES and DM II RR by the other covariates was explored through mediation analysis. The mediation analysis took results from the univariate model for SES as a baseline, and examined the percentage change to the estimated coefficient for SES when each of the other covariates was added to the model to form a bivariate model. A change of more than 10% was considered indicative of potential mediation.

### Dealing with missing data

Three imputation methods that may be appropriate for spatial analysis of health survey data and are considered in this study include:

- Mean imputation. This method substitutes each missing observation with the mean of the non-missing observations for each particular covariate.
- Imputation using a multivariate normal (MVN) prior distribution for covariate data. This method estimates the correlations between covariates in the model and uses these covariate relationships to predict missing observations based on the non-missing observations for each region.
- Imputation using a CAR prior distribution for each covariate. This method estimates the spatial correlation for each covariate individually, and uses these spatial relationships to estimate missing observations for each covariate based on non-missing observations in neighbouring regions.

The appropriateness of each of these methods depends on the particular application. Here we evaluate these alternative methods in the context of the case study.

### Imputation methods

A cross-validation approach was used to compare the accuracy of three imputation methods in producing estimates close to observed values. Results from mean imputation were compared to results from imputation using multivariate normal and conditional autoregressive prior distributions. The aim of imputation was to improve the model in terms of a) estimating unobserved covariate information based on known covariate information, and b) estimating associations between DM II RR and covariates included in the model.

Six of the seven covariates included in models had missing data and for five of these this was substantial. Of the 71 Queensland LGAs included in this analysis, data were missing for three LGAs (4%) for proportion aged 45 years and older. Data were missing for 28 LGAs (39%) for four covariates: proportion overweight/obese, proportion daily smokers, proportion with insufficient physical activity, and proportion with adequate fruit intake. For proportion with adequate vegetable intake, data were missing for 32 LGAs (45%), including the 28 LGAs with missing data for other covariates.

The common practice of removal of cases with missing values would have resulted in an unacceptable reduction of the data (45%) of cases removed) and potential bias in the results. Imputation of the missing data was considered instead.

Methods for each of the three imputation approaches are detailed below:

1. Mean imputation. For covariates *j* = 1 *to* 6 for the six covariates requiring imputation for missing values and regions *i* = 1 *to w*_*j*_ where *i* are the regions with missing values and *w*_*j*_ are the total number of regions with values to be imputed for covariate *j*, each missing observation for each covariate was replaced with the mean of the non-missing observations for that covariate. This preserves the mean of the observed data but does not account for correlations among variables and underestimates standard deviation of data after imputation.
2. Imputation using a MVN prior distribution for covariates. A variance-covariance matrix was fit to account for variance of and correlations between each of the seven explanatory variables. An inverse Wishart distribution with inverse variances of 0.01 for all covariates, and inverse covariances of 0.001 between covariates was fit as a prior to the variance-covariance matrix. Posterior estimates of the missing data were then obtained based on the observed data. The form of the multivariate normal prior for the design matrix ***X***, containing covariate data was:

where ***M*** is a vector of mean values and ***Σ*** is a variance-covariance matrix with an inverse Wishart distribution; ie. the inverse of ***Σ*** has a Wishart distribution with parameters ***ψ***, *ν*. The Wishart distribution is a generalisation to multiple dimensions of the chi-squared distribution.

(3) Imputation using CAR prior distributions for covariate data for each covariate *j*. The expected value of a missing datum for region *i* was estimated using a Normal prior distribution around the average of the observed values for that covariate in neighbouring regions. This approach borrows strength from neighbouring regions and accounts for spatial correlation between neighbours in covariate values.

The form of CAR priors fit separately for each covariate was:


where for region *i*, *V*_*i*_|*V*_*k*_ is the correlated random effect given the correlated random effect in neighbouring region *k*, *μ*(*v*_*k*_) is average correlated random effect for all adjacent neighbours, *m*_*i*_ is the number of such neighbours, and  is the conditional variance of *V*. The same neighbours are defined as for equation (3).

Multiple rounds of cross-validation were used to assess how accurately the imputation models performed on an independent dataset. Cross-validation was performed using only the 39 Queensland LGAs with full information for all seven covariates. For each of ten rounds of cross-validation, the data were split into two complementary subsets: 90% of data (35 LGAs) were randomly selected to form the training dataset, and the remaining 10% (4 LGAs) formed the test dataset. A conundrum with cross-validation approaches for spatial data is that estimation is improved by including as much data as possible in the training dataset, thus our decision to include 90% of data in the training dataset. However, the consequence is that a small sample remains for testing the results of imputation against the observed values. Due to this difficulty, imputation results for this case study should be treated with caution – however, the methodology is applicable to other datasets with larger sample sizes.

For each round of cross-validation, the observed covariate information in the test dataset were assigned missing values for the purpose of imputation. Each of the three imputation models were fit to the training dataset and used to impute values for the test dataset. The imputed values were then compared to observed values for each covariate in the test dataset by computing the root mean squared error (RMSE) for each covariate. For covariates *j* = 1 *to* 6 for the six covariates requiring imputation for missing values and *i* = 1 *to w*_*j*_ where *i* are the regions with missing values and *w*_*j*_ are the total number of regions with values to be imputed for covariate *j*, the RMSE for each covariate *j* is computed as follows for an estimated parameter *x*:


where  is the imputed value for region *i* and covariate *j*, and *x*_*ij*_ is the observed value of the parameter for region *i* and covariate *j*. The overall RMSE was computed giving each covariate equal weighting; however an alternative possibility would be to give each missing value equal weighting.

Imputation using MVN and CAR priors were compared to each other with respect to bias, defined as the average difference between predicted values and the mean observed value for a covariate, adjusted by the size of that mean observed value. By definition, mean imputation assigns the mean observed value for each covariate to missing values, resulting in a bias of zero. For each value imputed by MVN or CAR prior for covariates, bias was computed as follows:


where  is the predicted value for region *i* and covariate *j*, and  is the mean observed value across all observations for covariate *j*.

The overall bias was computed as an average of biases for each covariate as follows:


For imputed missing values, the following information was collected and compared between MVN and CAR prior imputation methods:

1. RMSE – this measures how close imputed values are to the observed values for each covariate and overall;
2. Mean bias – averaged over imputed observations for each covariate and overall. This measures whether or not a particular imputation method tends to overestimate or underestimate values overall for a particular dataset;
3. Mean width of 95% credible intervals for bias – averaged over imputed observations for each covariate and overall. In Bayesian statistics, a 95% credible interval (CI) is a two-tailed interval containing 95% of the posterior probability distribution. A wider interval for a particular imputation method indicates that estimated values fluctuated from the expected value of zero bias to a greater degree than an imputation method with a narrower interval.
4. Proportion of 95% CIs including zero bias for each covariate and overall. A smaller proportion for a particular imputation method indicates that more of the intervals missed the expected value of zero for bias.

The imputation method providing the smallest overall RMSE and bias was selected for further analyses.

### Sensitivity analysis

Sensitivity analysis was used to evaluate the impact of different priors on the posterior estimates of the model. The models were compared in terms of posterior estimates of the coefficients, and posterior inferences. The following priors were considered for both Binomial and Poisson models:

1. CAR priors fit to both covariate data ***X*** and correlated random effects, *S*_*i*_; Gamma(1,0.01) priors for precisions of components of *U*_*i*_ (Baseline model)
2. Gamma(1,0.01) priors for precisions of all components of the vectors ***β*** and *U*_*i*_; CAR priors for *S*_*i*_
3. Uni(0.01,5) priors for standard deviations of all components of the vectors ***β*** and *U*_*i*_; CAR priors for *S*_*i*_
4. Half normal priors, N(0,0.0625)I(0,) for standard deviation of components of *U*_*i*_; gamma(1,0.01) priors for precisions of components of ***β***; CAR priors for *S*_*i*_
5. Log normal priors, N(0,4) for standard deviation of components of log(*U*_*i*_); gamma(1,0.01) priors for precisions of components of ***β***, CAR priors for *S*_*i*_

More detailed information on priors included in the sensitivity analyses is provided in Table 1.

**Table 1:** **Prior distributions used for parameters in Sensitivity analysis**

Parameter	Model 1	Parameter	Model 2	Parameter	Model 3	Parameter	Model 4	Parameter	Model 5
α	N(0,0.01)	α	N(0,0.01)	α	N(0,0.01)	α	N(0,0.01)	α	N(0,0.01)
β_j_;j = 1,…,7	CAR(1/Ƭ_βj_,R)	β_j_;j = 1,…,7	N(0,1/ Ƭ_βj_)	β_j_;j = 1,…,7	N(0,σ^2^_βj_)	β_j_;j = 1,…,7	N(0,1/ Ƭ_βj_)	β_j_;j = 1,…,7	N(0,1/ Ƭ_βj_)
U_i_;i = 1,…,N	N(α,1/Ƭ_U_)	U_i_;i = 1,…,N	N(α,1/ Ƭ_U_)	U_i_;i = 1,…,N	N(α,σ^2^_U_)	U_i_;i = 1,…,N	N(α,1/ Ƭ_U_)	U_i_;i = 1,…,N	N(α,1/ Ƭ_U_)
S_i_;i = 1,…,N	CAR(1/Ƭ_S_,R)	S_i_;i = 1,…,N	CAR(1/Ƭ_S_,R)	S_i_;i = 1,…,N	CAR((σ^2^_S_,R)	S_i_;i = 1,…,N	CAR(1/Ƭ_S_,R)	S_i_;i = 1,…,N	CAR(1/Ƭ_S_,R)
Ƭ_βj_	Ga(1,0.01)	Ƭ_βj_	Ga(1,0.01)	σ_βj_	U(0.01,5)	Ƭ_βj_	Ga(1,0.01)	Ƭ_βj_	Ga(1,0.01)
Ƭ_U_	Ga(1,0.01)	Ƭ_U_	Ga(1,0.01)	σ_U_	U(0.01,5)	σ_U_	N(0,0.0625)I(0,)	log(σ_U_)	N(0,4)
Ƭ_S_	Ga(1,0.01)	Ƭ_S_	Ga(1,0.01)	σ_S_	U(0.01,5)	Ƭ_S_	Ga(1,0.01)	Ƭ_S_	Ga(1,0.01)

α = intercept, j = covariates 1 to 7, β_j_ = vector of coefficients for covariates 1 to 7, i = Local Government Areas (LGAs) 1 to 71, U_i_ = uncorrelated residual error for LGAs 1 to 71, S_i_ = correlated residual error for LGAs 1 to 71, Ƭ_βj_ = vector of precisions for covariate coefficients, Ƭ_U_ = vector of precisions for uncorrelated residual error, Ƭ_S_ = vector of precisions for correlated residual error, σ_βj_ = vector of standard deviations for covariate coefficients, σ_U_ = vector of standard deviations for uncorrelated residual error, σ_S_ = vector of standard deviations for correlated residual error, Ga = Gamma distribution, U = Uniform distribution, CAR = CAR normal prior centred around zero, denoted CAR(variance, adjacency neighbourhood weight matrix), R = adjacency neighbourhood weight matrix with diagonal entries equal to number of neighbours; ie. *R*_*ii*_ = *m*_*i*_.

Results of sensitivity analysis were compared across models in terms of posterior means and 95% credible intervals of coefficient values, size of residual errors and DIC and significance of included covariates. Covariates were defined to be significantly associated with outcomes if the 95% credible interval of their coefficient did not include zero.

## Results

The results of our evaluation of the described imputation methods in the context of the case study are presented in this section.

### Descriptive analysis

Of the 71 Queensland LGAs included in this analysis, SES data were available for all LGAs. DM II prevalence data were missing for four smaller LGAs, three of which were also missing data for proportion over 45 years of age. These four LGAs also had missing data for other covariates apart from SES. Overall, data were missing for 28 LGAs (39%) for four covariates: proportion overweight/obese, proportion daily smokers, proportion with insufficient physical activity, and proportion with adequate fruit intake. For proportion with adequate vegetable intake, data were missing for 32 LGAs (45%), including the 28 LGAs with missing data for other covariates. The reason for missing lifestyle data for these 28 LGAs is that they had a sample size smaller than 60 in the Queensland self-reported health status survey and were not reported due to potential inaccuracy of results.

SES ranged from 1 to 7 across Queensland LGAs with mean 3.8 (standard deviation (SD) 1.8). Of observed values, the mean proportion over 45 years of age was 35% (SD 8%), mean proportion overweight or obese was 62% (SD 6%), mean proportion of daily smokers was 19% (SD 5%), mean proportion with insufficient physical activity was 49% (SD 7%), mean proportion with adequate fruit intake was 54% (SD 5%) and mean proportion with adequate vegetable intake was 12% (SD 4%).

Of the 71 LGAs, 22 (31%) had missing covariate information for 50% or more of their immediate neighbours. Of the 28 LGAs with missing information for all self-reported lifestyle covariates, 14 (50%) also had missing covariate information for 50% or more of their immediate neighbours.

Pearson’s correlation estimates returned an absolute value greater than 0.2 among 52% (11/21) of covariate pairs among the seven explanatory variables, indicating reasonably highly correlated covariate data. This motivates the investigation of a multivariate imputation approach, but the presence of substantial structured missing data supports the possible preference for mean imputation.

### Imputation

Mean imputation was found to have the lowest overall RMSE (32.5) for this dataset. The RMSE values for each covariate separately and overall, for each of the three imputation methods, are summarised in Table 2. Imputation using CAR priors for the covariates had the second lowest overall RMSE, of 46.1 from both Poisson and Binomial GLMMs. Imputation using MVN produced the overall highest RMSE, 71.1 from Poisson and 72.7 from Binomial GLMM.

**Table 2:** **Comparison of imputation methods by root mean squared error** (**RMSE**) **and bias from cross**-**validation**

Covariate	N missing	RMSE, mean (sd)					Average bias		Average width of CI		% of CIs including zero bias	
		Mean imputation	MVN		CAR prior							
			Poisson	Binomial	Poisson	Binomial	MVN	CAR prior	MVN	CAR prior	MVN	CAR prior
% over 45yrs of age	3	49.7	108.7 (21.2)	109.6 (19.5)	46.3 (17.8)	46.2 (17.9)	0.012	0.041	0.897	0.345	100%	100%
% Overweight/obese	28	26.3	73.7 (22.3)	73.1 (20.6)	45.4 (18.5)	45.4 (18.4)	0.016	0.081	0.413	0.200	100%	75%
% Daily smokers	28	25.8	53.2 (18.1)	54.4 (19.3)	49.8 (21.1)	49.8 (21.0)	0.153	0.271	1.144	0.640	100%	68%
% Insufficient physical activity	28	36.7	90.7 (37.5)	91.4 (37.1)	67.0 (41.3)	67.1 (41.3)	0.048	0.047	0.535	0.246	100%	93%
% Adequate fruit intake	28	34.4	67.4 (14.3)	68.6 (14.7)	37.5 (22.7)	37.6 (22.7)	0.069	0.052	0.382	0.221	100%	91%
% Adequate vegetable intake	32	21.9	39.1 (18.5)	39.2 (18.4)	30.4 (19.6)	30.6 (19.9)	0.157	0.185	1.144	0.973	100%	94%
Overall	-	32.5	71.1 (11.4)	72.7 (11.1)	46.1 (11.7)	46.1 (11.7)	0.076	0.113	0.752	0.438	100%	87%

*RMSE* = root mean squared error, sd = standard deviation, *MVN* = Multivariate normal imputation, CAR prior = conditional autoregressive prior imputation; CI = 95% credible interval.

**Figure 1:**
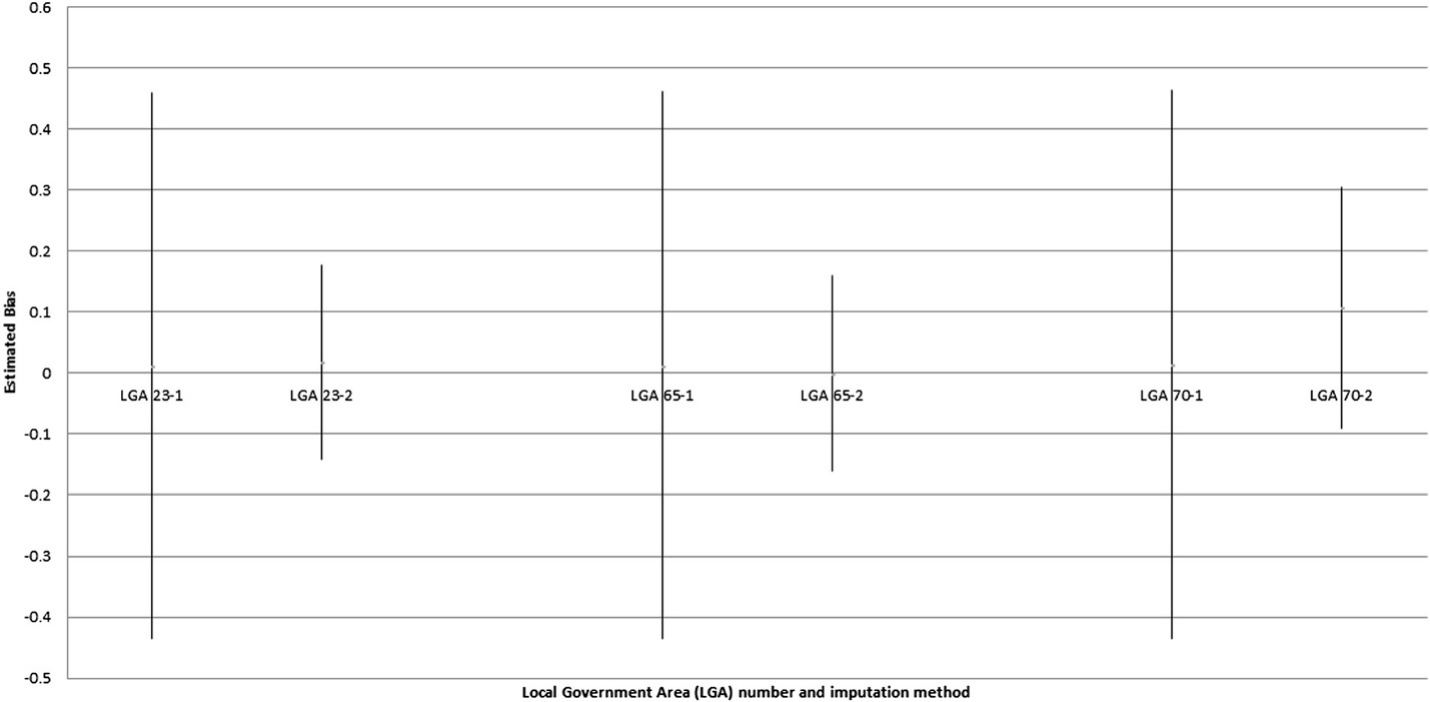
**Bias for estimated** **% over 45 years for Local Government Areas** **(LGAs)** **with missing data,** **by 1. Multivariate normal imputation,** **and 2. Conditional autoregressive (** **CAR)** **priors for covariates;** **e.g. LGA 23–1 indicates multivariate normal imputation for LGA number 23 and LGA 23–2 indicates imputation with CAR priors for covariates for LGA number 23.** LGA = Local Government Area).

Bias statistics for each covariate separately and overall are summarised in Table 2. The overall average bias from the imputation methods was largest for imputation using CAR priors (0.11) and smallest for MVN and mean imputation (estimated 0.08 for MVN and zero for mean imputation by definition). MVN imputation produced greater uncertainty of bias compared with imputation using CAR priors (average width of 95% credible interval (CI) was 0.75 and 0.44 respectively overall). Imputation using MVN consistently produced 95% CIs that included a bias value of zero, whereas only 87% of 95% CIs from imputation using CAR priors included a bias value of zero. A graphical comparison of estimate bias distribution between MVN and CAR prior imputation methods for one covariate, the proportion over 45 years of age, is provided in Figure 1. Bias plots for other covariates are available in the Additional file 1.

As the imputation method providing both the smallest RMSE and bias for this dataset, mean imputation was selected and was adopted for further analyses, including sensitivity analysis. Although there is some built-in circularity favouring mean imputation as an unbiased method of imputation by definition, overall it appears an appropriate choice for this dataset given a) it produced estimates that were closest to observed estimates, and b) the other two methods produced significant bias for two covariates in particular: proportion of daily smokers and proportion with adequate vegetable intake.

### Sensitivity analysis

Mean estimates of selected parameters resulting from Binomial and Poisson GLMMs with different priors are displayed in Table 3. Each of the GLMMs included in sensitivity analysis produced similar coefficient estimates and resulted in the same conclusions.

**Table 3:** **Estimates for selected parameters from models included in sensitivity analysis**: **mean** (**95**% **credible intervals**)

Binomial	α	β _1_	β _2_	β _3_	β _4_	β _5_	β _6_	β _7_	σ _S_^2^	σ _U_^2^	DIC
1	-2.158	-0.194	0.009	-0.004	0.008	-0.008	-0.013	-0.005	0.013	0.073	667
	(-2.368,-1.963)	(-0.240,-0.143)	(-0.001,0.020)	(-0.019,0.012)	(-0.010,0.027)	(-0.022,0.006)	(-0.036,0.011)	(-0.033,0.022)			
2	-2.147	-0.197	0.009	-0.005	0.008	-0.007	-0.015	-0.004	0.013	0.074	667
	(-2.415,-1.911)	(-0.253,-0.129)	(-0..002,0.021)	(-0.024, 0.013)	(-0.013, 0.027)	(-0.023, 0.008)	(-0.040,0.008)	(-0.031,0.025)			
3	-2.158	-0.194	0.01	-0.005	0.008	-0.007	-0.014	-0.005	0.012	0.079	666
	(-2.374,-1.939)	(-0.248,-0.1416)	(-0.002,0.021)	(-0.022,0.011)	(-0.010,0.026)	(-0.022,0.006)	(-0.038,0.007)	(-0.032,0.022)			
4	-2.155	-0.194	0.009	-0.003	0.008	-0.007	-0.014	-0.004	0.012	0.076	668
	(-2.384,-1.951)	(-0.242,-0.138)	(-0.002,0.0196)	(-0.020,0.016)	(-0.010,0.026)	(-0.022,0.008)	(-0.038,0.009)	(-0.030,0.023)			
5	-2.203	-0.183	0.008	-0.004	0.008	-0.006	-0.013	-0.003	0.012	0.080	666
	(-2.451,-1.953)	(-0.242,-0.122)	(-0.004,0.020)	(-0.026,0.015)	(-0.013,0.029)	(-0.024,0.011)	(-0.037,0.009)	(-0.032,0.027)			
**Poisson**											
1	0.641	-0.181	0.009	-0.005	0.007	-0.006	-0.014	-0.005	0.012	0.062	671
	(0.440,0.854)	(-0.232,-0.134)	(-0.001,0.020)	(-0.022,0.011)	(-0.009,0.024)	(-0.020,0.008)	(-0.035,0.008)	(-0.030,0.022)			
2	0.615	-0.174	0.008	-0.004	0.008	-0.005	-0.011	-0.004	0.012	0.061	671
	(0.434,0.816)	(-0.223,-0.133)	(-0.002,0.018)	(-0.020,0.012)	(-0.008,0.026)	(-0.018,0.007)	(-0.031,0.009)	(-0.028,0.022)			
3	0.649	-0.183	0.009	-0.004	0.008	-0.006	-0.013	-0.003	0.012	0.067	670
	(0.413,0.864)	(-0.236,-0.125)	(-0.002,0.020)	(-0.025,0.014)	(-0.010,0.025)	(-0.021,0.008)	(-0.036,0.011)	(-0.029,0.025)			
4	0.651	-0.184	0.009	-0.004	0.007	-0.007	-0.013	-0.005	0.012	0.065	672
	(0.422,0.883)	(-0.240,-0.129)	(-0.002,0.020)	(-0.023,0.014)	(-0.012,0.025)	(-0.021,0.007)	(-0.036,0.010)	(-0.031,0.022)			
5	0.646	-0.182	0.009	-0.003	0.008	-0.007	-0.012	-0.006	0.011	0.066	670
	(0.441,0.888)	(-0.244,-0.134)	(-0.002,0.020)	(-0.021,0.016)	(-0.012,0.025)	(-0.022,0.008)	(-0.034,0.009)	(-0.030,0.022)			

α = intercept, β_1_ = coefficient for socio-economic status, β_2_ = coefficient for % over 45 years of age, β_3_ = coefficient for % overweight/obese, β_4_ = coefficient for % daily smokers, β_5_ = coefficient for % insufficient physical activity, β_6_ = coefficient for % adequate fruit intake, β_7_ = coefficient for % adequate vegetable intake, σ_S_^2^ = variance of correlated residual error, σ_U_^2^ = variance of uncorrelated residual error, *DIC* = Deviance Information Criteria.

Prior distributions used in models 1–5 are summarised in Table 1.

## Results

SES was found to be the only variable associated with DM II RR based on the Poisson models and prevalence based on the Binomial models, from both univariate and multivariate models. Of the other covariates included in the models, none were found to be significantly associated with DM II outcomes. From the baseline Poisson model (model 1), each one unit increase in SES was estimated to decrease the log(relative risk) of DM II by 0.18 (95% credible interval 0.13 to 0.23).

Mediation analysis did not find a significant mediating effect (defined by a change of 10% or more to the SES coefficient) between SES and DM II RR by any of the other covariates included in this study.

### Geographic variation

**Figure 2:**
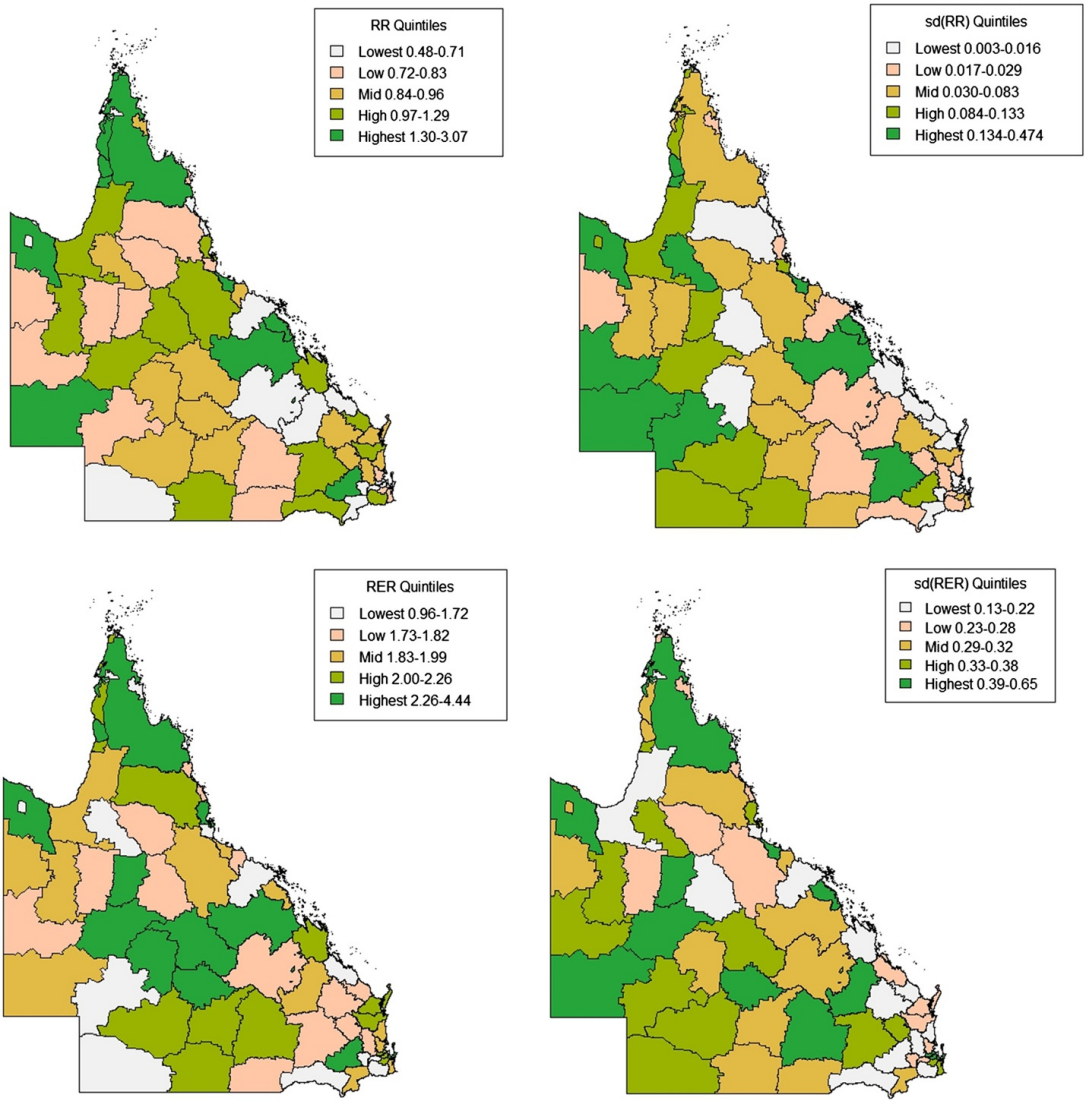
**Estimated Relative Risk** (**RR**) **and Relative Excess Risk** **(RER)** **of type 2 diabetes for Queensland Local Government Areas.** RR = relative risk, sd = standard deviation, RER = relative excess risk).

Spatially smoothed relative risks (RR) and relative excess risks (RER) and corresponding standard deviations and 95% credible intervals were obtained from the Poisson GLMMs with mean imputation and CAR priors fit to covariate data. The estimated RR of DM II varied between study regions from 0.48 (Isaac Regional) to 3.07 (Cherbourg Aboriginal Shire), indicating a six-fold variation (3.07/0.48 = 6.4) across regions. RER varied from 0.96 for Napranum Aboriginal Shire to 4.44 for Burke Shire. The distribution of RR and RER by quintiles from highest to lowest are displayed in Figure 2 along with their standard deviation. The size of estimated RR and RER for each region does not appear to be associated with the size of uncertainty for those regions.

The LGAs with the five smallest and five highest RR, RER and standard deviation for RR and RER are ranked in Table 4. 80% of the regions in the top five for large RR also were in the top five for large RER, indicating that they are most at risk for DM II occurrence even after accounting for the influence of regional risk factors.

**Figure 3:**
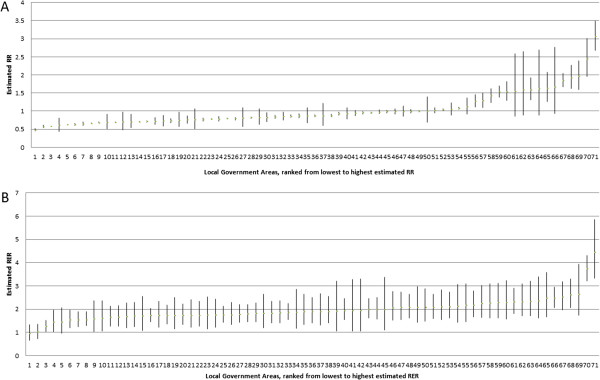
**Ranked Relative Risk** **(A)** **and Relative Excess Risk** **(B)** **for Local Government Areas with 95%** **credible intervals.** RR = Relative Risk, RER = Relative Excess Risk).

Figure 3 ranks regions in order of low to high RR (A) and RER (B) respectively with 95% CIs. As may be expected, regions with missing covariate data tended to have wider 95% CIs compared with regions with observed data.

**Table 4:** **Top 5 LGAs for Relative Risk** (**RR**), **Relative Excess Risk** (**RER**) **and uncertainty for Relative Risk and Excess Relative Risk**

Smallest estimated RR		Smallest sd(RR)		Smallest estimated RER		Smallest sd (RER)	
LGA	Estimated RR	LGA	sd(RR)	LGA	Estimated ERR	LGA	sd(RER)
34	0.480	8	0.003	47	0.962	67	0.125
16	0.573	28	0.005	32	1.021	10	0.150
8	0.580	44	0.006	67	1.255	30	0.152
24	0.611	60	0.006	34	1.442	32	0.161
28	0.624	38	0.007	24	1.452	57	0.168
Largest estimated RR		Largest sd(RR)		Largest estimated RER		Largest sd(RER)	
LGA		LGA	sd(RR)	LGA	Estimated RER	LGA	sd(RER)
63	1.857	12	0.269	51	2.535	68	0.566
35	1.933	41	0.445	35	2.627	70	0.575
51	1.966	65	0.452	68	2.645	41	0.580
12	2.450	70	0.465	18	3.738	23	0.587
18	3.073	23	0.474	12	4.442	12	0.647

*RR* = Relative Risk, *RER* = Relative Excess Risk, *sd* = standard deviation.

## Discussion

Our study describes an evaluation of three different imputation methods that are applicable to missing health survey data for spatial analysis. Choice of imputation method depends upon the particular application and is not necessarily the most complex method. In the application for this case study, simple imputation with the mean value of each missing covariate value was found to provide the most accurate prediction of missing values in this dataset, based on the statistical measures described.

In this application, mean imputation was found to be more appropriate than imputation with CAR priors using spatial correlation of covariate data to impute missing values. For this dataset, this could be due to the large proportion of missingness for some covariates relative to a small number of neighbours for certain regions, providing insufficient observed data from neighbouring regions. These different imputation methods may perform comparatively differently in datasets with smaller proportions of missing data.

Simple mean imputation was also found to be far more accurate in this case study than fitting a multivariate normal distribution to covariates to impute missing data in this dataset, despite empirical evidence of high correlation between many covariate pairs. This is likely due to the pattern of missingness, as LGAs tended to have either complete data for all covariates, or missing data for six covariates (proportion overweight/obese, daily smokers, aged over 45 years, proportion with insufficient physical activity, and sufficient fruit and vegetable intake). Moreover, missingness was related to population size of LGAs, as less-populated LGAs did not have covariate data from the Queensland self-reported health status survey. Thus data were not missing at random in this dataset. Multivariate normal imputation may provide more accurate prediction of missing values in datasets with missingness at random as well as high correlations between covariate pairs.

Our sensitivity analysis provides evidence that choice of priors, from non-informative to more informative choices, did not affect results from the spatial analysis of this case study. Fitting of Binomial and Poisson models produced similar findings with similar goodness of fit as measured by DIC. This supports the estimates of DM II RR for each LGA and evidence that SES is strongly associated with DM II risk in this region. The sensitivity analysis described in this paper is readily applicable to spatial analysis of other health datasets.

Several studies have examined geographic variation in DM II in the US, UK and Europe, however, less is known about regional variation and associated regional risk factors in Australia. Similar to other studies, our analysis shows marked geographic variation in DM II relative risk [7, 9, 11–18]. Just within Queensland, our study estimates a six-fold difference in DM II relative risk. Similar to findings from other spatial studies, we found lower socioeconomic status to be strongly associated with increased risk of DM II [9, 11, 13, 14].

Contrary to findings from Green et al., we did not find the proportion of daily smokers to be associated with DM II risk [11]. In comparison with risk models reporting BMI to be associated with DM II risk at an individual-level, we did not find the proportion of residents overweight or obese to be associated with DM II risk at a regional level in this dataset [8]. We examined the association of obesity (BMI ≥ 30kg/m^2^) and overweight (25kg/m^2^ ≤ BMI < 30kg/m^2^) with DM II RR separately in univariate models and neither were found to be significant within this geographic region. However, our study categorised BMI into broad overweight and obese categories whereas the risk models considered raw BMI scores. Findings may differ for spatial analyses of DM II risk in other regions.

Strengths of our study include that we were able to evaluate the performance of three different imputation approaches using methodology which is immediately applicable to other regions and health datasets outside the application of the case study reported. Within our case study, we were able to evaluate the geographical variation in DM II RR across Queensland and identify regions of high risk, and regional factors associated with DM II risk, accounting for missing data. We used Bayesian methods to fit hierarchical models accounting for different sources of uncertainty, to evaluate the association of geographical covariates with DM II RR. Spatial smoothing was performed, accounting for correlation between neighbouring regions and mitigating the effects of random measurement error. In addition, we were able to select the most accurate imputation method for this dataset and check the accuracy of results through sensitivity analysis.

Limitations of our study include the presence of significant missing data, small sample sizes for test datasets in cross-validation, that diabetic counts were based on notification data with unknown measurement bias, and that region-level lifestyle data was based on self-report that is not objectively measured. Thus results should be interpreted with caution.

Although spatial modelling of DM II relative risk at a smaller region level such as Statistical Local Area (SLA) may have resulted in relative risk information at a finer level, the difficulty is that lifestyle information is not available at this level and cannot be assessed for contribution to DM II risk. Furthermore, we expect less uncertainty from variation in notification rates when data is aggregated to a larger regional level.

## Conclusions

In conclusion, we present a method for selection of an appropriate imputation method among alternative choices suited to spatial health survey data with varying patterns and amounts of missingness. Missing data is a common problem with spatial health data, and appropriate choice of imputation method depends upon the particular application. As discovered for the case study considered here, choice of imputation method may not always be the most complex one. However in some cases, utilising other information such as spatial correlation in data or correlation between covariates may be appropriate for the purposes of imputation. Selection of an appropriate imputation method allows a more complete analysis of geographic risk factors for disease at a regional level, with the potential to inform resource allocation and public policy, and reduce the burden of disease to the community.

This case study provides evidence of a six-fold difference in geographical variation in DM II RR across Queensland LGAs, and indicates that socio-economic status is strongly associated with DM II risk. Our results indicate that a geographically targeted approach to managing DM II may be effective, and highlight regions most in need of additional services to manage DM II. The methodology used in this study is applicable to spatial analyses of diabetes in other regions, as well as other diseases, and has the potential to provide useful information for management and resource allocation decisions.

## Electronic supplementary material

Additional file 1: **Bias for estimates for each covariate for regions with missing data.** (DOCX 53 KB)
