# Supplementary material for: Missing in space: an evaluation of imputation methods for missing data in spatial analysis of risk factors for type II diabetes
Source: Int J Health Geogr. 2014 Nov 20;13:47. doi: 10.1186/1476-072X-13-47 (PMC4287494; doi:10.1186/1476-072X-13-47)
Supplement: Supplementary file 1 — Additional file 1: Bias for estimates for each covariate for regions with missing data. (DOCX 53 KB) [file 12942_2014_616_MOESM1_ESM.docx]

Appendix

1. Bias for estimated % overweight/obese for LGAs with missing data, by 1. Multivariate normal imputation, and 2. CAR priors for covariates; eg. LGA 23-1 indicates multivariate normal imputation for LGA number 23 and LGA 23-2 indicates imputation with CAR priors for covariates for LGA number 23.

2. Bias for estimated % daily smokers for LGAs with missing data, by 1. Multivariate normal imputation, and 2. CAR priors for covariates; eg. LGA 23-1 indicates multivariate normal imputation for LGA number 23 and LGA 23-2 indicates imputation with CAR priors for covariates for LGA number 23.

3. Bias for estimated % with insufficient physical activity for LGAs with missing data, by 1. Multivariate normal imputation, and 2. CAR priors for covariates; eg. LGA 23-1 indicates multivariate normal imputation for LGA number 23 and LGA 23-2 indicates imputation with CAR priors for covariates for LGA number 23.

4. Bias for estimated % with adequate fruit consumption for LGAs with missing data, by 1. Multivariate normal imputation, and 2. CAR priors for covariates; eg. LGA 23-1 indicates multivariate normal imputation for LGA number 23 and LGA 23-2 indicates imputation with CAR priors for covariates for LGA number 23.

5. Bias for estimated % with adequate vegetable consumption for LGAs with missing data, by 1. Multivariate normal imputation, and 2. CAR priors for covariates; eg. LGA 23-1 indicates multivariate normal imputation for LGA number 23 and LGA 23-2 indicates imputation with CAR priors for covariates for LGA number 23.
